# Rapid Determination of RNA Modifications in Consensus Motifs by Nuclease Protection with Ion-Tagged Oligonucleotide Probes and Matrix-Assisted Laser Desorption Ionization Mass Spectrometry

**DOI:** 10.3390/genes13061008

**Published:** 2022-06-02

**Authors:** Madeline E. Melzer, Jonathan V. Sweedler, Kevin D. Clark

**Affiliations:** 1 Department of Biochemistry, University of Illinois at Urbana-Champaign, Urbana, IL 61801, USA; mmelzer2@illinois.edu (M.E.M.); jsweedle@illinois.edu (J.V.S.); 2 Beckman Institute for Advanced Science and Technology, University of Illinois at Urbana-Champaign, Urbana, IL 61801, USA; 3 Department of Chemistry, University of Illinois at Urbana-Champaign, Urbana, IL 61801, USA

**Keywords:** RNA modifications, matrix-assisted laser desorption ionization mass spectrometry (MALDI-MS), m^6^A consensus motif, ion-tagged oligonucleotides (ITOs), RNA modification stoichiometry, nuclease protection

## Abstract

The reversible and substoichiometric modification of RNA has recently emerged as an additional layer of translational regulation in normal biological function and disease. Modifications are often enzymatically deposited in and removed from short (~5 nt) consensus motif sequences to carefully control the translational output of the cell. Although characterization of modification occupancy at consensus motifs can be accomplished using RNA sequencing methods, these approaches are generally time-consuming and do not directly detect post-transcriptional modifications. Here, we present a nuclease protection assay coupled with matrix-assisted laser desorption ionization mass spectrometry (MALDI-MS) to rapidly characterize modifications in consensus motifs, such as GGACU, which frequently harbor N6-methyladenosine (m^6^A). While conventional nuclease protection methods rely on long (~30 nt) oligonucleotide probes that preclude the global assessment of consensus motif modification stoichiometry, we investigated a series of ion-tagged oligonucleotide (ITO) probes and found that a benzylimidazolium-functionalized ITO (ABzIM-ITO) conferred significantly improved nuclease resistance for GGACU targets. After optimizing the conditions of the nuclease protection assay, we applied the ITO and MALDI-MS-based method for determining the stoichiometry of GG(m^6^A)CU and GGACU in RNA mixtures. Overall, the ITO-based nuclease protection and MALDI-MS method constitutes a rapid and promising approach for determining modification stoichiometries of consensus motifs.

## 1. Introduction

Newly discovered roles for post-transcriptional modifications in regulating protein translation [1,2] and RNA metabolism [3,4] have rendered these unusual chemical alterations to the primary structure of RNA increasingly important targets for quantitative analysis. Across virtually all types of coding and non-coding RNAs, approximately 170 unique RNA modifications have been discovered to date that range in complexity from methylation and isomerization to isopentenylation and conjugation with cellular metabolites [5]. Modifications can be deposited, removed, and recognized by “writer”, “eraser”, and “reader” proteins [3,6], respectively, contributing to dynamic fluctuations in the cellular RNA modification landscape. This landscape, or “epitranscriptome,” is responsive to external stimuli and is involved in regulating translation during heat shock [7], oxidative stress [8], methylation stress [9], and experience-dependent plasticity in the central nervous system [10,11,12]. Dramatic variations in RNA modification identity and abundance have also been characterized in small clusters of cells and even single cells [13,14,15,16], further driving the demand for methods that are capable of rapidly characterizing and quantifying modified RNAs.

RNA modifications are often deposited by enzymatic writers at predictable sequence motifs [17,18,19]. For example, a METTL3–METTL14 complex methylates adenosine in GGACU consensus motifs to form N6-methyladenosine (m^6^A) [20,21]. In addition to being one of the most abundant RNA modifications comprising ~0.5% of total adenosines in human cell lines [22], m^6^A can also be enzymatically removed from these consensus sites to mediate gene expression [23,24,25]. In normal function, dynamic methylation in GGACU motifs influences developmental programs, learning and memory [10,11], and axon regeneration following injury [26]. Dysregulation of m^6^A and its writer and eraser enzymes has been linked to tumorigenicity of cancer cells [27], neurodegenerative disease [28], and addiction/reward learning [29]. Characterization of modification occupancy across these functionally important motifs is therefore highly valuable and is typically accomplished using immunoprecipitation or chemical derivatization coupled with RNA-seq approaches [22,30,31,32,33,34]. However, these methods typically require time-consuming sample preparation steps, have limited quantitative capabilities, and do not directly detect RNA modifications, which may lead to false positive and/or false negative signals.

Direct detection and quantification of RNA modifications is often accomplished using mass spectrometry (MS) techniques [35,36,37,38,39]. Typical MS-based strategies involve digestion of full-length RNA molecules into smaller fragments using sequence-selective enzymes such as RNase T1, followed by detection, identification, and quantification of the resulting fragments by liquid chromatography-tandem mass spectrometry (LC-MS/MS). While there are many advantages to LC-MS/MS, a disadvantage of LC-MS/MS methods is that each sample requires analysis times that may limit sample throughput. Matrix-assisted laser desorption ionization-mass spectrometry (MALDI-MS) is a high-throughput alternative that involves the rapid ablation of samples with a laser to generate ions for MS detection. The MALDI-MS system can directly detect an RNA modification that results in a change in mass-to-charge ratio (*m*/*z*) [40,41,42,43]. Another advantage of MALDI-MS is that it is a soft ionization technique that permits the analysis of intact nucleic acids up to ~2000 nt in length [44]. MALDI-MS has also been applied for the characterization of RNase T1 digests to map RNA modifications [40,45,46]. For targeted analysis of RNA modifications in specific regions of an RNA molecule, MALDI-MS oligonucleotide hybridization probes can be combined with single-strand-specific nucleases to remove untargeted segments of RNA prior to analysis [47]. These nuclease protection assays are particularly attractive when complex samples containing a large variety of RNAs are analyzed, since only the target RNA sequence is protected from degradation. However, nuclease protection methods are typically only implemented with hybridization probes ~20–50 nt in length to maximize base-pairing interactions between the probe and the target sequence. As a result, short RNA sequences like GGACU consensus motifs cannot be readily analyzed for their modification status using conventional oligonucleotide probes.

In order to improve hybridization with target nucleic acids, chemical functionalization of oligonucleotide probes can provide enhanced melting temperatures and selectivity for complementary sequences [48]. Recently, thiol-ene click reactions were used to synthesize ion-tagged oligonucleotides (ITOs) with alkyl and benzylimidazolium substituents tethered to the 3′ terminus of a thiolated DNA probe [49,50]. By applying ITO probes for hybridization with DNA targets, the selectivity for complementary sequences relative to mismatches can be improved. Because ITOs have previously been investigated for high-efficiency capture of nucleic acids for DNA diagnostic applications [51,52,53], these probes may also prove useful for hybridization with and characterization of modified RNAs.

In this study, we report a MALDI-MS-based nuclease protection assay that utilizes ITO hybridization probes to protect GGACU and GG(m^6^A)CU targets, thus facilitating the analysis of modification stoichiometry at these sites. A series of alkylimidazolium and benzylimidazolium ITOs were investigated to enhance the stability of short target RNA sequences during digestion with single-strand-specific mung bean nuclease (MBN), revealing that a benzylimidazolium-functionalized ITO (ABzIM-ITO) maximized the liberation of the GGACU motif from full-length RNA. After optimizing the ITO-based nuclease protection assay, we investigated the effect of sequence context on the detection of GGACU motifs. Unlike conventional DNA probes, we found that the ABzIM-ITO showed no preference for terminal or internal placement of the GGACU motif sequences across RNAs, suggesting that the ITO hybridization probe is suitable for the unbiased evaluation of RNA modification occupancy at these sites. As a proof of concept, the ITO and MALDI-MS-based approach was applied for determining modification stoichiometry in heterogeneous pools of modified and unmodified RNAs. Overall, our results demonstrate that nuclease protection assays can be improved by using ITO hybridization probes and MALDI-MS to rapidly characterize RNA modification stoichiometries within short RNA sequences.

## 2. Materials and Methods

### 2.1. Reagents and Materials

All oligonucleotides were purchased from Integrated DNA Technologies (Coralville, IA, USA) or Dharmacon (Lafayette, CO, USA) and used without further purification (Table S1). Mung bean nuclease and mung bean nuclease reaction buffer were purchased from New England Biolabs (Ipswich, MA, USA). Tris(2-carboxyethyl)phosphine (TCEP) hydrochloride, triethylamine, acetic acid, urea, 3-hydroxypicolinic acid (3-HPA), acetonitrile, and C18 ZipTip Pipette Tips with 0.6 µL bed volume were purchased from Millipore-Sigma (St. Louis, MO, USA). A 40% acrylamide/bis-acrylamide (19:1) solution, ammonium persulfate, tetramethylethylenediamine (TEMED), and Microseal B adhesive were purchased from Bio-Rad (Hercules, CA, USA). Milli-Q water (Millipore, Burlington, MA, USA) was used for the preparation of all solutions. All imidazolium salts used in this study were received as gifts from J. L. Anderson (Iowa State University) and stored in a desiccator until use.

### 2.2. Ion-Tagged Oligonucleotide Synthesis

The ITOs used in this study have been reported previously and were prepared by thiol-ene click reactions using published procedures [49,50]. Briefly, a thiolated oligonucleotide was reduced with 40 nmol TCEP for every 4 nmol of thiolated oligonucleotide and incubated at room temperature (20–23 °C) for 45 min. After reduction, 1.4 µL of the oligonucleotide solution was deposited in the center of a well in a 96-well plate. A 0.5 μL aliquot of 800 nM allylimidazolium salt solution prepared in acetonitrile (ACN) was then added, and the solution mixed via pipet. The microwell was then sealed with UV-transparent tape. The seal was punctured with a 21 G needle for venting, and the well was subsequently purged with nitrogen gas delivered at a gentle flow to avoid turbulence in the reaction liquid. After briefly purging the well (~3 s), the needles were removed, and the well resealed with UV-transparent tape. The plate was then placed on a cooling fan in a dark environment. A handheld UV lamp set to 365 nm was then placed on top of the plate for 2 h at room temperature.

After the reaction, the mixture was diluted with 2 µL of nuclease-free water and 4 µL 7 M urea and subjected to denaturing polyacrylamide gel electrophoresis (PAGE). Unreacted thiolated oligonucleotide was separated from the ITO product using a 15% polyacrylamide gel prepared with 7 M urea. The gel was run at 130 V in tris-borate–EDTA buffer for ~45 min, and the bands were visualized by UV shadowing at 254 nm. The ITO band (i.e., the slower migrating band) was excised, transferred to a 1.5 mL tube, and crushed with a pipet tip. The ITO was eluted from the gel with 40 µL of nuclease-free water overnight, and the resulting ITO concentration determined by NanoDrop spectrophotometry.

### 2.3. Nuclease Protection and Purification of Target RNA

In a 0.2 mL PCR tube, 1 µL of 10X mung bean nuclease reaction buffer and 10–50 pmol of target RNA were combined with ITO probe (2:1 probe to target ratio) and water to a total volume of 9.5 µL. The oligonucleotides were annealed using the following temperature program: 65 °C for 3 min, hold at 4 °C. Next, 0.5 µL of mung bean nuclease (10,000 U mL^−1^) was added, and the reaction was digested at 15 °C for 30 min.

Following digestion, the reaction mixture was diluted with 10 µL of 0.1 M triethylammonium acetate at pH 7 (TEAA). C18 pipet tips were pre-conditioned with 3 × 10 µL washes of 50/50 ACN/H_2_O followed by 3 × 10 µL washes of 0.1 M TEAA. The reaction solution was then loaded by repeatedly drawing and dispensing the solution 10 times. The C18 pipet tip was washed with 3 × 10 µL of 0.1 M TEAA. The oligonucleotides were eluted in a clean microcentrifuge tube containing 5 µL of 50/50 ACN/H_2_O by drawing and dispensing 20 times.

### 2.4. Matrix-Assisted Laser Desorption Ionization Mass Spectrometry

A 0.5 µL aliquot of the sample was deposited on an MTP-384 polished steel target (Bruker), immediately followed by 1.0 µL of 0.5 M 3-HPA matrix prepared in 50/50 ACN/water. The sample was allowed to dry completely at room temperature.

MALDI-MS was performed using a Bruker ultrafleXtreme MALDI-TOF mass spectrometer operated in reflectron-positive mode over a 700–5000 *m*/*z* range with a Bruker smartbeam-II laser set to “Ultra” (diameter of ~70 μm) and 83% power. The instrument was calibrated using a peptide calibration standard mixture (Bruker). Each spectrum resulted from 10,000 total laser shots generated across 10 manually selected positions, where each position was sampled with 1000 shots at a frequency of 1000 Hz. The instrument specific settings included pulsed extraction time of 120 ns, accelerating voltage of 25 kV, extraction voltage of 22.65 kV, lens voltage of 6.8 kV, and reflector voltage of 26.4 kV.

### 2.5. Data Analysis

Mass spectra were processed using Bruker flexAnalysis 3.4, and peaks were manually annotated and verified with Mongo Oligo Mass Calculator v2.06 (http://rna.rega.kuleuven.be/masspec/mongo.htm, accessed 1 April 2022). For each sample, the stability ratio (SR) was calculated using Equation (1):

(1) $$SR=1-\frac{\sum_{i=1}^{n}I_{f1}+I_{f2}+\ldots +I_{fn}}{I_{target}}$$

where *I_f_*_1_, *I_f_*_2_, and *I_fn_* are the peak intensities for the 1st, 2nd, and *n*th most intense degradation fragments with signal-to-noise (SN) ratios ≥10, and *I_target_* is the peak intensity for the intact target sequence (GGACU or GG(m^6^A)CU) with SN ≥ 10 following digestion of the sample with single-strand-specific MBN.

## 3. Results and Discussion

### 3.1. ITO-Facilitated Nuclease Protection Coupled with MALDI-MS for the Characterization of RNA Consensus Motifs

**Figure 1:**
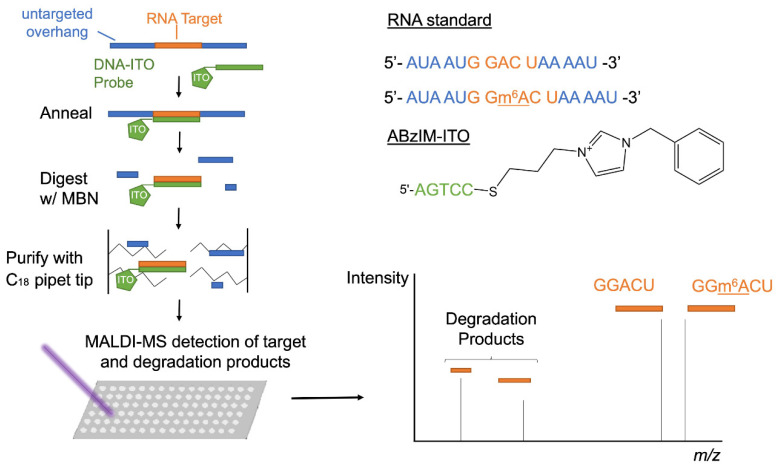
Schematic showing the workflow for the ITO-based nuclease protection assay including ITO probe–target annealing, MBN digestion, sample purification, and MALDI-MS detection of the liberated target sequence (GGACU or GG(m^6^A)CU) and target degradation fragments.

Nuclease protection coupled with MALDI-MS is a high-throughput approach for characterizing RNA modifications that relies on an oligonucleotide probe complementary to the RNA sequence of interest and a single-strand-specific nuclease that cleaves the unhybridized regions of RNA molecules. Nuclease protection assays have thus far only been implemented with relatively long oligonucleotide probes (~20–50 nt) that cannot be used to globally interrogate the modification status of small consensus motifs across the transcriptome [54,55]. In order to improve the effectiveness of nuclease protection assays for short RNA target sequences, we investigated ion-tagged oligonucleotide (ITO) probes that have been previously shown to enhance the hybridization selectivity for target nucleic acids [49,50]. We used thiol-ene click reactions to tether allylimidazolium salts bearing butyl (ABIM), octyl (AOIM), decyl (ADIM), and benzyl (ABzIM) groups to the 3′ terminus of short thiolated oligonucleotides (5 nt) complementary to an m^6^A consensus motif (Figure S1 and Table S2). This series of ITOs was implemented in a nuclease protection workflow using the single-strand-specific nuclease, MBN, coupled with MALDI-MS for the detection of the m^6^A consensus motif (GGACU) installed within a 15 nt synthetic RNA standard (Figure 1).

**Figure 2:**
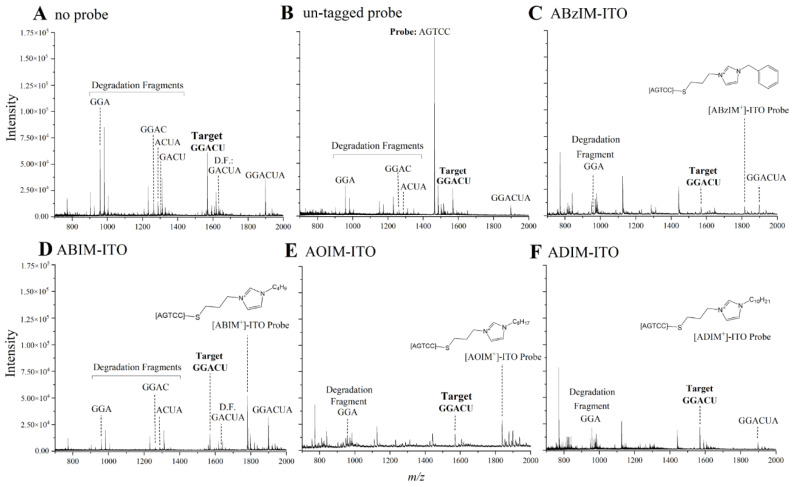
Representative MALDI mass spectra following MBN digestion of a 15-mer RNA standard treated with (**A**) no hybridization probe, (**B**) a conventional DNA probe, (**C**) ABzIM-ITO, (**D**) ABIM-ITO, (**E**) AOIM-ITO, and (**F**) ADIM-ITO. Peaks in each mass spectrum were manually annotated to indicate the probe, the liberated GGACU target, and any detectable target degradation fragments derived from the unsuccessful protection of GGACU.

Our first goal was to identify ITO probes that minimized the amount of degradation fragments produced from the target GGACU sequence while maximizing the amount of target sequence liberated from the RNA standard. ITO probes were annealed with the RNA standard (2:1 probe–target ratio) and incubated for 30 min with MBN at 30 °C according to the nuclease manufacturer’s instructions. After purifying the samples with C18 pipet tips, the samples were spotted with 3-HPA and subjected to MALDI-MS detection. The MALDI-MS platform permitted the rapid (~10–20 s per sample) and simultaneous detection of the consensus motif as well as the degradation fragments resulting from unsuccessful protection of the motif (Figure 2A–F). For each sample, the stability ratio (SR) was calculated (Equation (1)) to assess the abundance of intact GGACU target relative to its degradation fragments. Higher SRs indicate superior protection of the target sequence from nuclease degradation. Although MALDI-MS ionization efficiencies may differ for RNA fragments of different lengths (e.g., intact target versus degradation fragments), the SR is an assessment of the relative intensities of these peaks, and thus, subtle differences in ionization of these fragments would not impact trends in SRs. As expected, intense degradation fragment peaks and low-intensity GGACU peaks were observed for samples without hybridization probe, resulting in the lowest SRs of the samples tested. For the ITOs, SRs generally increased with increasing chain length for the alkylimidazolium probes (Table S3), possibly due to enhanced dispersion forces between the probe and the target that improve strand association and/or to increasing steric bulk of the probe–target duplex to facilitate nuclease protection. The ABzIM-ITO resulted in the highest SRs, perhaps due to favorable π-π stacking interactions between the benzylimidazolium group and adjacent nucleobases, which is similar to the enhanced stability of nucleic acid duplexes previously observed upon attachment of cyanine dye labels to the terminus of an oligonucleotide probe [56]. Based on these results, we pursued further optimization of the method with the ABzIM-ITO.

### 3.2. Optimization of ITO-Based Nuclease Protection Conditions

**Figure 3:**
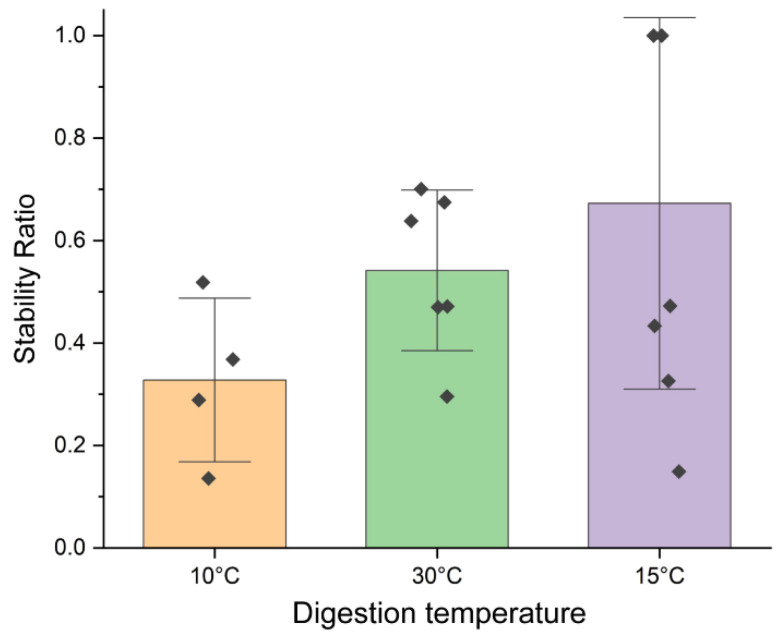
Stability ratios calculated for nuclease protection assays using the ABzIM-ITO probe and the 15-mer RNA standard containing an internal GGACU motif using different digestion temperatures. Individual data points are represented by black diamonds. Error bars represent ±1 SD.

We then investigated the effect of different digestion temperatures to identify conditions that both facilitated nuclease function and stabilized the ABzIM-ITO probe and the target RNA duplex. We tested the nuclease protection assay using 15 °C and 10 °C for the digestion step to compensate for the low melting temperature of the probe–target duplex (~10 °C) [57]. SRs for these conditions as well as for the 30 °C digestion temperature were calculated (Figure 3), revealing that digestion at 15 °C trended toward the highest SR and thus the best nuclease protection. These results can be explained by the higher temperature (30 °C) causing denaturation of the probe-target duplex, which renders the target sequence vulnerable to MBN digestion. For the lower temperature digestion (10 °C), we hypothesized that lower SRs would be obtained due to the low activity of the nuclease, resulting in a lower abundance of the target GGACU sequence liberated from the RNA standard. Despite 15 °C being a lower temperature than what is suggested as optimal for MBN assays, it appears that a 15 °C digestion temperature is suitable for nuclease protection assays for short RNA sequences, such as the m^6^A consensus motif studied here.

**Figure 4:**
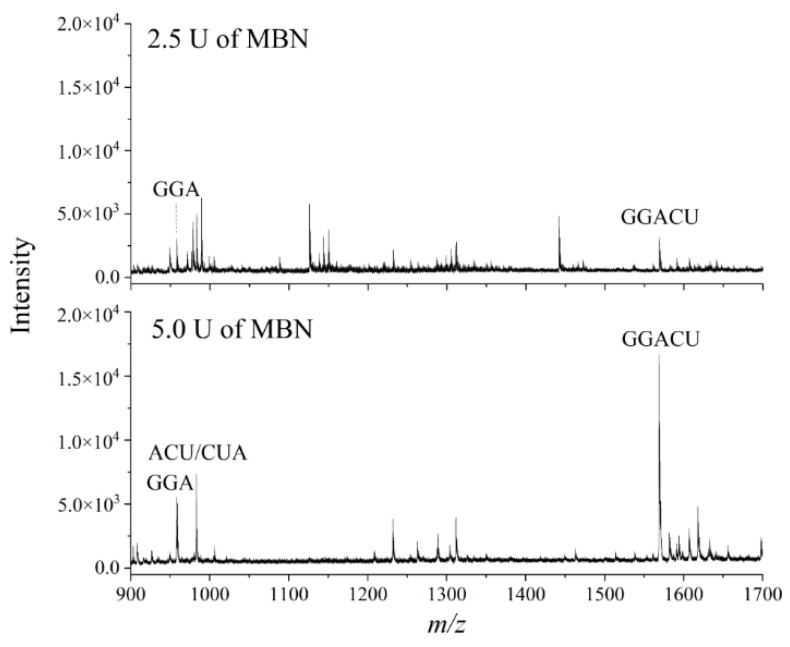
Representative mass spectra resulting from MALDI-MS following nuclease protection assays using the ABzIM-ITO and a 15-mer RNA standard with an internal GGACU motif and different amounts of single-strand-specific MBN.

Next, we compared SRs obtained when performing digestion with different amounts of MBN in the sample mixture. We rationalized that since degradation fragments had already been observed using 5 U of MBN, decreasing the amount of MBN to 2.5 U might improve the SR. In contrast to our expectations, we found that the lesser amount of MBN resulted in a lower signal intensity for the target peak, which in turn produced a substantially lower SR (Figure 4). This result was likely due to the inefficient liberation of the GGACU target from the 15-mer RNA as a consequence of fewer units of MBN in the reaction mixture. We therefore selected 5 U of MBN as optimal for subsequent experiments.

### 3.3. Comparison of 3′ and 5′ Ion Tag Structures and Sequence Context of Consensus Motifs on Nuclease Protection

**Figure 5:**
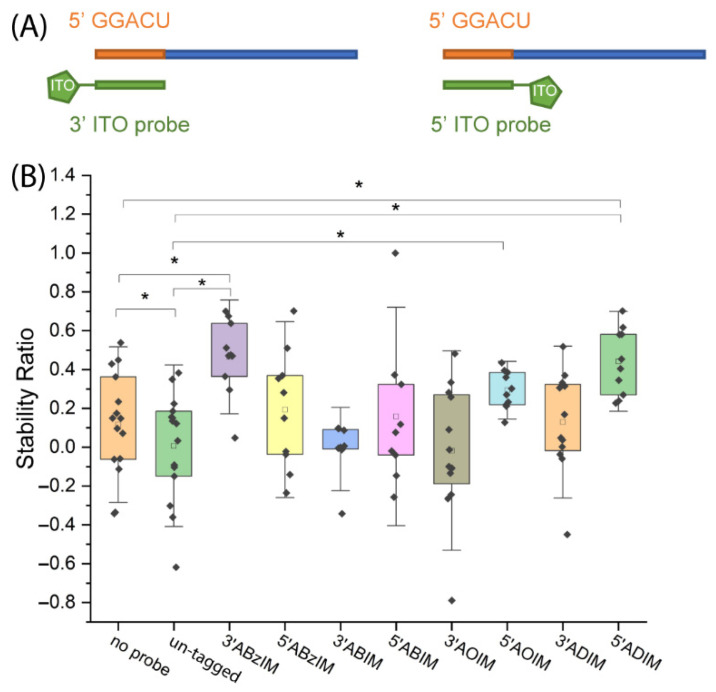
(**A**) Schematic representation of RNA target and 3′ or 5′ ITO probes. (**B**) Comparison of stability ratios obtained for ITOs with 3′ or 5′ functional groups when applied in a nuclease protection assay involving a 15-mer RNA standard with a 5′ terminal GGACU motif target. Within each box plot (25th to 75th percentile), the small square in each box represents the mean of the data. Individual data points are represented by black diamonds. Error bars are 1.5 SD, unpaired *t*-test, * *p* < 0.05.

We then investigated the effect of installing the ion tag moiety on the 3′ or 5′ end of the ITO probe. Since the ITO group was appended to the 3′ end of the ABzIM-ITO probe in previous experiments, we anticipated that the enhanced target stability during nuclease digestion was due to π-π stacking interactions with the 5′ terminal guanosine of the GGACU motif. To test this hypothesis, a 15-mer RNA standard possessing a 5′ terminal GGACU motif was subjected to nuclease protection with either a 3′ or a 5′ functionalized ABzIM-ITO probe (Figure 5A). Significantly higher SRs were once again observed for the 3′ ABzIM-ITO compared to an un-tagged probe and no-probe samples, whereas the 5′ ABzIM-ITO showed no significant effect on improving nuclease protection under the same conditions (Figure 5B). These results aligned with our hypothesis that because a pyrimidine terminated the GGACU motif on the 3′ end, π-π stacking interactions with the benzyl group of the 5′ ABzIM-ITO were diminished compared to the situation in which a purine base was present.

To further investigate this result, we compared SRs for a series of 3′ alkylimidazolium ITO probes (ABIM, AOIM, ADIM) whose functional groups cannot engage in stacking interactions. The SRs obtained from 3′ alkyl-functionalized ITOs were not significantly different from protection by an un-tagged probe or the no-probe control (Figure 5B), supporting the conclusion that the benzyl group of the ABzIM-ITO facilitates enhanced protection due to favorable π-π stacking with adjacent purine nucleobases. We then investigated the effect of placing alkylimidazolium ion tag moieties on the 5′ terminus of the ITO. ITOs with long alkyl chains (C_8_ and C_10_) produced significantly higher SRs compared to the un-tagged and/or no-probe control samples (Figure 5B). The 5′ ABIM-ITO showed no significant increase in nuclease protection compared to either control sample. These results may be due to steric bulk provided by the long alkyl groups of the AOIM-ITO and ADIM-ITO probes that impeded the MBN degradation of the target.

**Figure 6:**
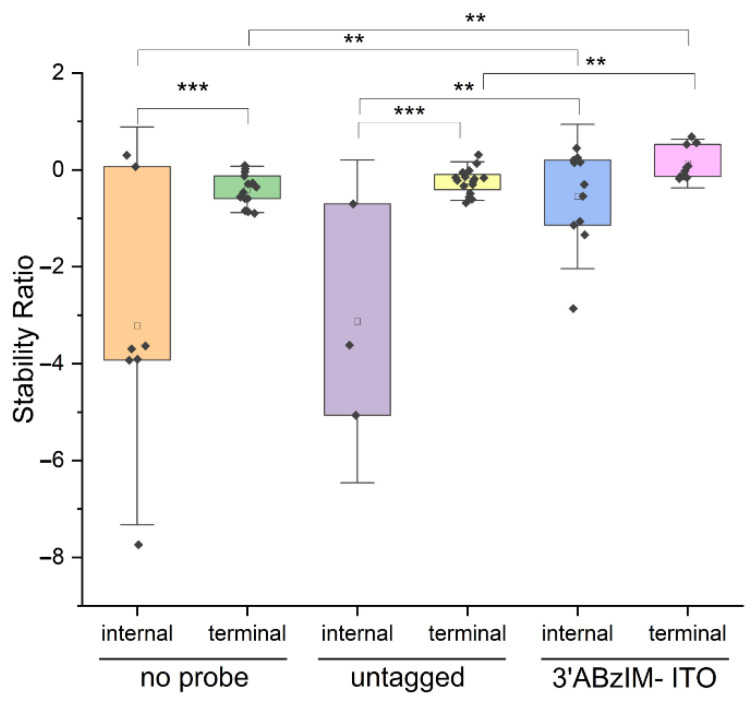
Comparison of stability ratios for nuclease protection assays involving 15-mer RNA standards with either a 5′ terminal or an internal GGACU motif. Assays were conducted without a probe, with an un-tagged probe, or with the ABzIM-ITO. Within each box plot (25th to 75th percentile), the small square represents the mean of the data. Individual data points are represented by black diamonds. Error bars are 1.5 SD, unpaired *t*-test, ** *p* < 0.01, *** *p* < 0.001.

Another motivation for studying the effect of the sequence context of the consensus motif on the nuclease protection assay is that RNA modifications can be deposited both internally and near the 5′ termini of RNA biopolymers [58,59,60]. To determine whether the ABzIM-ITO showed bias toward protecting GGACU motif sequences depending on sequence context, we compared the SRs obtained from ABzIM-ITO-mediated protection of 15-mer RNA standards designed with either 5′-terminal GGACU or internal GGACU motifs. For the un-tagged probe, bias toward protection of the 5′ terminal GGACU motif was observed, illustrated by significantly higher SRs for terminal GGACU compared to the internal motif (Figure 6). In contrast, we found no difference in stability ratios when using the ABzIM-ITO based on the sequence context of the consensus motif. These results indicate that the ITO probe is suitable for unbiased global analysis of m^6^A occupancy at these sites.

### 3.4. Evaluation of m^6^A Stoichiometry in the GGACU Consensus Motif Using ITO-Based Nuclease Protection and MALDI-MS

**Figure 7:**
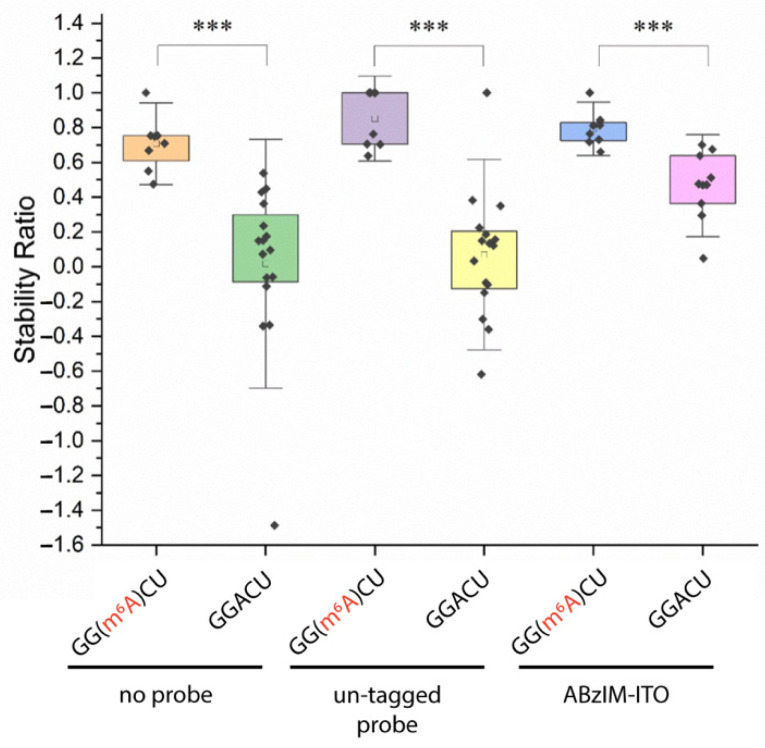
Comparison of stability ratios obtained from nuclease protection assays using 15-mer RNA standards possessing an m^6^A-modified or unmodified 5′ terminal consensus motif (GG(m^6^A)CU or GGACU). Within the box plots (25th to 75th percentile), the small square indicates the mean. Individual data points are represented by black diamonds. Error bars are 1.5 SD, unpaired *t*-test, *** = *p* < 0.001.

We then aimed to apply the ITO and MALDI-MS method for the determination of RNA modification stoichiometry at the consensus motifs. First, we designed 15-mer RNA standards with either internal GGACU or GG(m^6^A)CU sequences and asked whether the 3′ ABzIM-ITO probe would confer nuclease resistance to the m^6^A-modified RNA. After accounting for the changes in the *m*/*z* of the target and degradation peaks originating from the modified RNA (Table S1), we found that SRs were significantly higher for the methylated RNA compared to the unmodified RNA in all samples, suggesting that the m^6^A modification is more resistant to MBN digestion (Figure 7). These results imply that oligonucleotides containing m^6^A are less favorable substrates for MBN degradation, which appears to counterbalance the generally destabilizing effects of m^6^A on base pairing [61]. We then investigated the precision of the method near the limit of quantification (LOQ, SN = 10), which consisted of a mixture of 15-mer RNA standards bearing a 5′ modified or unmodified consensus motif. Using 10 pmol of each modified and unmodified RNA, the method resulted in signal-to-noise ratios of 13.7 and 16.5 and percent relative standard deviations (RSDs) of 20.8 and 23.7% for the liberated GGACU and GG(m^6^A)CU targets, respectively (Table S4). These RSDs agree with previously reported values for quantitative analysis of 5-mer RNA standards by MALDI-MS [62]. The limit of detection (LOD) for GG(m^6^A)CU was calculated from three times the noise of a blank analysis and was 1.8 pmol.

**Figure 8:**
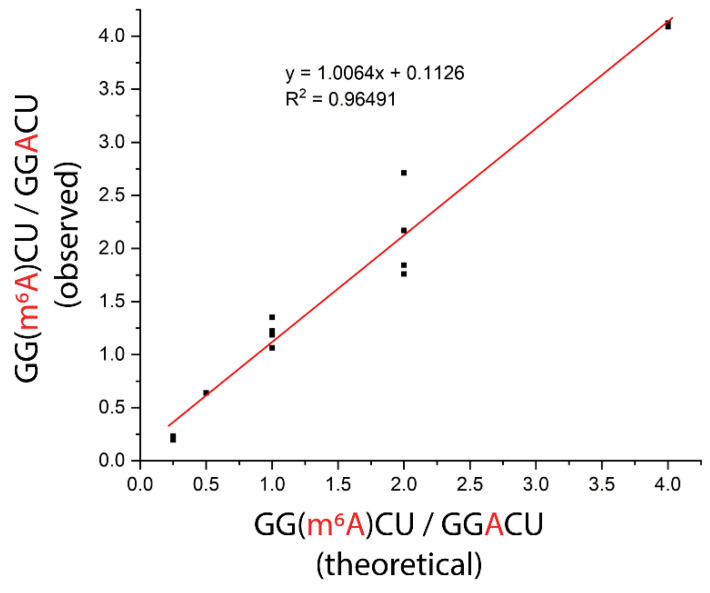
Evaluation of modification stoichiometry for an m^6^A consensus motif (GGACU) in samples prepared with different ratios of modified-to-unmodified RNA. Each sample consisted of controlled amounts of 15-mer RNA standards with either a 5′ GGACU or a 5′ GG(m^6^A)CU motif. The total amount of RNA was 100 pmol in all samples. The 3′ ABzIM-ITO was used for nuclease protection prior to MALDI-MS. Replicates for different stoichiometries were as follows: 1:4 (*n* = 2), 1:2 *(n* = 2), 1:1 (*n* = 4), 2:1 (*n* = 4), and 4:1 (*n* = 2).

As a proof of concept, we applied the ITO and MALDI-MS-based nuclease protection assay for the determination of modification stoichiometry using a sample mixture that contained both modified and unmodified sequences. While keeping the total amount of RNA in the sample constant, we tested different ratios of modified to unmodified RNAs in the sample, including 1:4, 1:2, 1:1, 2:1, and 4:1, which span the range of stoichiometries previously reported for m^6^A sites in mRNAs from cell lines determined using chemical derivatization and RNA-seq [22]. We subjected each sample to the nuclease protection assay using the 3′ ABzIM-ITO probe and calculated the ratio of signal intensities for the modified and unmodified target sequences. The experimentally determined ratios of modified to unmodified consensus motif present in the sample were in good agreement with the amounts added to the sample and showed a linear relationship (R^2^ = 0.97) across the stoichiometries tested (Figure 8). Overall, these results show that the ITO-based nuclease protection assay coupled to MALDI-MS provides a promising alternative to RNA-seq platforms for the rapid determination of global RNA modification stoichiometries at consensus motifs.

## 4. Conclusions

In this study, we report a nuclease protection assay that leverages ITO hybridization probes and MALDI-MS to characterize RNA modification stoichiometries in a short consensus motif that endogenously harbors m^6^A (GGACU). While typical approaches for determining modification stoichiometries at these sites rely on RNA sequencing methods that include time-consuming library preparation steps and indirect detection by immunoprecipitation or chemical derivatization, the coupling of nuclease protection and MALDI-MS affords rapid analysis and direct detection of any RNA modification that alters the *m*/*z* of the resulting RNA sequence. This feature MS detection is important for the discovery of unknown RNA modifications or known modifications in unexpected sites. To improve nuclease protection assays for the analysis of short target sequences, we investigated a series of hybridization probes including alkylimidazolium- and benzylimidazolium-functionalized ITOs. We found that the ABzIM-ITO maximized the liberation of target GGACU motifs for detection by MALDI-MS, likely due to enhanced π-π stacking interactions with the target RNA sequence. Unlike conventional un-tagged hybridization probes, the ABzIM-ITO did not show any bias toward protection of internal or terminally positioned motif sequences, which is an important feature for the unbiased evaluation of modification stoichiometry across multiple sites in RNA molecules. When applied for the determination of modification stoichiometries in consensus motifs, the results from the ITO and MALDI-MS-based approach strongly agreed (R^2^ = 0.97) with the ratios of modified and unmodified RNAs added to the sample. In addition, since dozens of samples can be subjected to nuclease protection and purification within ~45 min, the detection methods for the resulting digest must have sufficient sample throughput to keep pace. While LC-MS of RNA oligonucleotides require ~30–60 min per RNA digest, the MALDI-MS analysis times reported herein are rapid and only require ~10–20 s per digest, thus providing a high-throughput alternative for characterizing modified and unmodified RNA fragments generated from nuclease protection.

This study establishes a foundation for subsequent investigations into global RNA modification stoichiometry in complex samples that contain numerous targeted and untargeted RNA sequences. One of the advantages of the nuclease protection assay is that it dramatically reduces the complexity of RNA sequences within a sample via single-strand-specific digestion, potentially allowing for the rapid analysis of modification stoichiometries even in total RNA samples. In our experiments, we found that the LOQ for this approach was 10 pmol of RNA molecules that each contained one motif sequence. We expect that further improvements in the LOQ of the method will allow the characterization of RNA modification events in successively smaller samples, such as subregions of the mammalian central nervous system. For example, with ~10^6^ neurons in the mouse hippocampus, ~10^5^–10^6^ mRNAs per cell, and an estimated ~3–5 m^6^A modification sites per transcript [24], a two-fold improvement in LOQ for the nuclease protection and MALDI-MS method would permit the characterization of modification stoichiometry within this functionally important brain region. One way to improve the detection limits of the assay includes the design of new hybridization probes that further stabilize short consensus motifs. The results we report herein indicate that π-π stacking interactions are useful for enhancing resistance toward single-strand-specific nucleases, providing a starting point for the design of hybridization probes that capitalize on these intermolecular forces. Additionally, the design of ITOs with longer complementary sequences (~20 nt) may also permit measurements of modification stoichiometry at single m^6^A sites in specific mRNA transcripts. The application of longer ITO hybridization probes would be particularly interesting for transcripts containing multiple m^6^A consensus motifs. While such an approach would be limited by transcript abundance, it is conceivable that high-abundance mRNAs would be assessable.

A limitation of our method is that it appears to have the best performance when a purine nucleobase is present at the 5′ terminus of the consensus motif. However, it is conceivable that consensus motifs bearing purine nucleobases at their 3′ ends could be sufficiently protected by a 5′-functionalized ITO probe, allowing adaptation of the ITO-based method for a variety of consensus motifs such as UGUAR (R denotes purine) for pseudouridine (Ψ) [17] or GUUCRA for N1-methyladenosine (m^1^A) [19]. Implementing the ITO-based nuclease protection assay for different RNA modifications will require consideration of the differences in hybridization efficiencies that may exist between modified RNAs and their unmodified counterparts.

## Data Availability

Data are available upon request to the corresponding author.

## Supplementary Materials

The following supporting information can be downloaded at: https://www.mdpi.com/article/10.3390/genes13061008/s1, Figure S1: Photograph of a denaturing gel after the electrophoretic separation of a series of ITO probes from their unreacted thiolated oligonucleotide counterparts. The top band in each lane is the ITO while the bottom band in unreacted thiolated oligonucleotide. ABzIM, ABIM, AOIM, and ADIM ITOs were generated from allylbenzylimidazolium, allylbutylimidazolium, allyloctylimidazolium, and allyldecylimidazolium salts, respectively, Table S1. The RNA standard sequences used in this study with the target sequence underlined. Theoretical and observed *m*/*z* values for the liberated target sequence are shown, as well as RNA fragments derived from nuclease digestion/unsuccessful protection of the GGACU motif. Table S2: Chemical structures and abbreviations of the un-tagged probe and ITOs used in this study as well as corresponding theoretical and observed *m*/*z* values. Table S3: Stability ratios for nuclease protection assays using different alkylimidazolium or benzylimidazolium ITO probes compared to a standard DNA oligo probe and no probe samples Table S4: Figures of merit for the nuclease protection method using 3′ ABzIM-ITO and MALDI-MS detection for characterization of modified or unmodified consensus motifs in a 15-mer RNA standard.
