# Supplementary material for: Rapid Determination of RNA Modifications in Consensus Motifs by Nuclease Protection with Ion-Tagged Oligonucleotide Probes and Matrix-Assisted Laser Desorption Ionization Mass Spectrometry
Source: Genes (Basel). 2022 Jun 2;13(6):1008. doi: 10.3390/genes13061008 (PMC9222981; doi:10.3390/genes13061008)
Supplement: Supplementary file 1 [file genes-13-01008-s001.zip › genes-1712702-supplementary/supplementary materials.pdf]

# Rapid Determination of RNA Modifications in Consensus Motifs by Nuclease Protection with Ion-Tagged Oligonucleotide Probes and Matrix-Assisted Laser Desorption Ionization Mass Spectrometry

Madeline E. Melzer,[1,2] Jonathan V. Sweedler,[1,2,3] and Kevin D. Clark,[2,3,]*

[1]Department of Biochemistry, University of Illinois at Urbana-Champaign, Urbana, Illinois 61801, United States

[2]Beckman Institute for Advanced Science and Technology, University of Illinois at Urbana-Champaign, Urbana, Illinois 61801, United States

[3]Department of Chemistry, University of Illinois at Urbana-Champaign, Urbana, Illinois 61801, United States

*Correspondence: clarkkd@illinois.edu

## TABLE OF CONTENTS

Figure S1.......S2
Table S1.......S3
Table S2.......S4
Table S3.......S5
Table S4.......S6

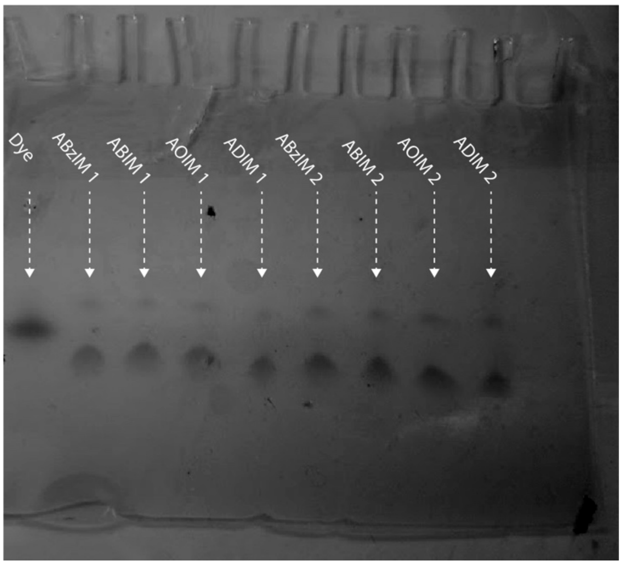


**Figure S1**. Photograph of a denaturing gel after the electrophoretic separation of a series of ITO probes from their unreacted thiolated oligonucleotide counterparts. The top band in each lane is the ITO while the bottom band in unreacted thiolated oligonucleotide. ABzIM, ABIM, AOIM, and ADIM ITOs were generated from allylbenzylimidazolium, allylbutylimidazolium, allyloctylimidazolium, and allyldecylimidazolium salts, respectively.

**Table S1.** The RNA standard sequences used in this study with the target sequence underlined. Theoretical and observed *m/z* values for the liberated target sequence are shown, as well as RNA fragments derived from nuclease digestion/unsuccessful protection of the GGACU motif.

| Sequence Name | Sequence (5' to 3') | *m/z* of target sequence (theoretical) | *m/z* of target sequence (observed) | *m/z* MBN degradation products (sequence, theoretical *m/z*, observed *m/z*) |
|---|---|---|---|---|
| 15mer RNA Internal Target | AUA AU<u>G GAC U</u>AA AAU | 1649.230 | 1649.383 | pAC U, 959.136, 959.148<br>pGAC, 998.158, 998.727<br>pAUG, 999.142<br>pUGG, 1015.137, 1015.501<br>pG GA, 1038.164, 1037.851<br>pACUA, 1288.188, 1288.039<br>pGACU, 1304.183, 1304.134<br>pG GAC, 1343.205, 1343.612<br>pAUGG, 1344.189, 1344.323<br>pGACUA, 1633.235<br>pAUGGA, 1673.241 |
| 15mer RNA Terminal Target | <u>GGA CU</u>A UAA UAA AAU | 1569.264 | 1569.204 | GGAC, 1263.239, 1262.996<br>GGA, 958.198, 958.070<br>pCUA/pACU, 959.136, 958.994<br>pGAC, 998.158,<br>pCUAU, 1265.161, 12<br>pACUA, 1288.188,<br>pGACU, 1304.183, 1304.605<br>pGACUA, 1633.235, 1632.832 |
| 15mer RNA Modified Terminal Target | <u>GGm$_6$A CU</u>A UAA UAA AAU | 1583.291 | 1583.022 | GGm6AC, 1277.266, 1277.403<br>GGm6A, 972.225, 972.483<br>pCUm6A, 959.136, 959.041<br>pm6ACU, 973.163, 972.884<br>pGm6AC, 1012.185,<br>pCUAU, 1265.161<br>pm6ACUA, 1302.215<br>pGm6ACU, 1318.21, 1318.490<br>pGm6ACUA, 1647.262, 1647.432 |

**Table S2**. Chemical structures and abbreviations of the un-tagged probe and ITOs used in this study as well as corresponding theoretical and observed *m/z* values.

| ITO Probe | Structure | Theoretical *m/z* | Observed *m/z* |
|---|---|---|---|
| Unmodified AGTCC | 5'-AGTCC-3' | 1463.30 | 1463.207 |
| ABzIM | 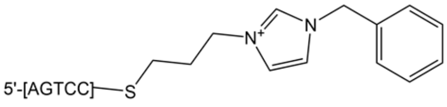 | 1815.40 | 1815.564 |
| ABIM | 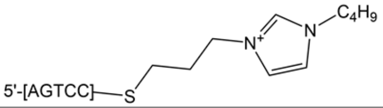 | 1781.42 | 1781.265 |
| AOIM | 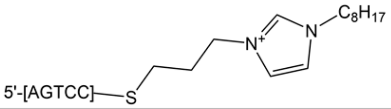 | 1837.48 | 1837.407 |
| ADIM | 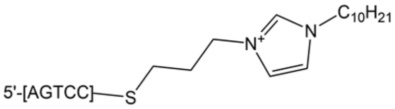 | 1865.51 | 1865.187 |

**Table S3**. Stability ratios for nuclease protection assays using different alkylimidazolium or benzylimidazolium ITO probes compared to a standard DNA oligo probe and no probe samples.

| probe | stability ratio (avg) | standard deviation |
|---|---|---|
| no probe | -0.281 | 0.620 |
| no tag | -0.192 | 0.335 |
| ABzIM-ITO | 0.409 | 0.222 |
| ABIM-ITO | -0.016 | 0.343 |
| AOIM-ITO | -0.320 | 0.363 |
| ADIM-ITO | 0.044 | 0.318 |

**Table S4**. Figures of merit for the nuclease protection method using 3' ABzIM-ITO and MALDI-MS detection for characterization of modified or unmodified consensus motifs in a 15-mer RNA standard.

| RNA target[a] | amount (pmol) | average signal-to-noise (n = 3) | average intensity (n = 3) | standard deviation (n = 3) | relative standard deviation (%, n = 3) |
|---|---|---|---|---|---|
| GGACU | 10 | 13.7 | 630.1 | 130.8 | 20.8 |
| GG($m^6$A)CU | 10 | 16.5 | 751.7 | 178.2 | 23.7 |

[a] 15-mer RNA standard with 5' consensus motif either modified or unmodified with $m^6$A.
