# Supplementary figures and images for: Rapid Determination of RNA Modifications in Consensus Motifs by Nuclease Protection with Ion-Tagged Oligonucleotide Probes and Matrix-Assisted Laser Desorption Ionization Mass Spectrometry
Source: Genes (Basel). 2022 Jun 2;13(6):1008. doi: 10.3390/genes13061008 (PMC9222981; doi:10.3390/genes13061008)

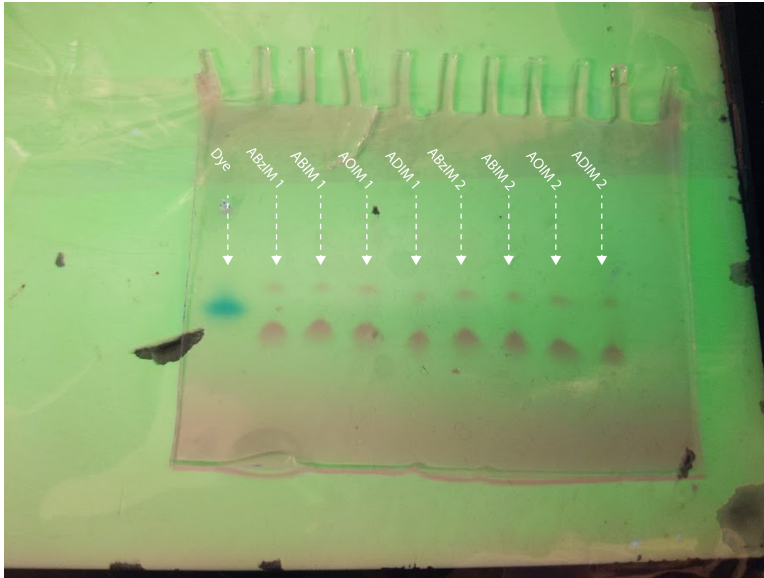

Supplement: Supplementary file 1 [file genes-13-01008-s001.zip › genes-1712702-supplementary/Figure S1 unfiltered-01.pdf]
